# Prevalence of intestinal parasitic infections and associated risk factors for hookworm infections among primary schoolchildren in rural areas of Nakhon Si Thammarat, southern Thailand

**DOI:** 10.1186/s12889-018-6023-3

**Published:** 2018-09-14

**Authors:** Chuchard Punsawad, Nonthapan Phasuk, Suchirat Bunratsami, Kanjana Thongtup, Parnpen Viriyavejakul, Sarawoot Palipoch, Phanit Koomhin, Somchok Nongnaul

**Affiliations:** 1 School of Medicine, Walailak University, Nakhon Si Thammarat, Thailand; 2 Tropical Diseases and Parasitic Infectious Diseases Research Group, Walailak University, Nakhon Si Thammarat, Thailand; 3 Department of Tropical Pathology, Faculty of Tropical Medicine, Mahidol University, Bangkok, Thailand; 4 Nopphitam Hospital, Nakhon Si Thammarat, Thailand

**Keywords:** Hookworms, Intestinal parasites, Soil-transmitted helminth, Schoolchildren, Southern Thailand

## Abstract

**Background:**

Soil-transmitted helminth (STH) infections are among the most common type of infections worldwide and are widely distributed in tropical areas. In rural areas of southern Thailand where most land is used for agriculture, children are at risk of acquiring parasites, especially STHs. Assessing the current situation regarding parasitic infection in these areas is a prerequisite for developing appropriate control measures. This study is aimed at determining the prevalence of intestinal parasitic infections, the intensity of STH infections and the associated risk factors among primary schoolchildren in Nopphitam District, Nakhon Si Thammarat Province, Thailand.

**Methods:**

A cross-sectional study involving 299 schoolchildren between 7 and 12 years of age was conducted between January and March 2016. A questionnaire administered by direct interviews was used to collect sociodemographic information and data on associated risk factors. Stool samples were processed using direct wet smears, formalin-ethyl acetate sedimentation concentration, and the modified Kato-Katz technique.

**Results:**

The overall prevalence of intestinal parasites among the 299 children was 16% (48 of 299), with 32 children infected with hookworms (10.7%), 10 with *Blastocystis hominis* (3.3%), seven with *Giardia intestinalis* (1.6%), one with *Enterobius vermicularis* (0.3%), and one with *Trichuris trichiura* (0.3%). The hookworm infection intensity, measured by the median eggs per gram (EPG) of stool, was 1200 EPG (Interquartile range (IQR): 360–3200). Most children had light-intensity hookworm infections, but two had heavy-intensity infections. When participants included in the sample were classified by age, children 10–12 years old demonstrated higher intestinal parasite prevalence than those aged 7–9 years (adjusted odds ratio (AOR) = 2.3, 95% CI: 1.1–4.9, *P* = 0.030). Inadequate handwashing before meals was statistically associated with hookworm infections (AOR = 2.3; 95% CI: 1.1–4.8, *P* = 0.037).

**Conclusions:**

This study highlights that hookworms are the most prevalent STH infection in the study area. Older age group (10–12 years) and inadequate handwashing before meals were statistically associated with hookworm infections. Accordingly, appropriate strategies and education on personal and environmental hygiene should be implemented. Moreover, the cost-effectiveness of mass drug administration in this area should be further investigated.

**Electronic supplementary material:**

The online version of this article (10.1186/s12889-018-6023-3) contains supplementary material, which is available to authorized users.

## Background

Soil-transmitted helminths (STHs) are intestinal worms that infect humans and are transmitted through contaminated soil. STH infection is considered a neglected tropical disease [1]. The main species that infect humans are roundworms (*Ascaris lumbricoides*), whipworms (*Trichuris trichiura*), and hookworms (*Necator americanus* and *Ancylostoma duodenale*) [2–4]. Approximately 2 billion people are infected with STHs worldwide [5]. The World Health Organization (WHO) has stated that school-aged children are particularly at risk for STH infection [5]. STH infection in children can lead to impaired physical, intellectual, and cognitive development [2, 6, 7]. Several risk factors are associated with STH transmission and infection, namely, age, poor environmental sanitation, poor personal hygiene, geography, socioeconomic status, and occupation [2, 3].

Several studies have reported the STH infection prevalence in schoolchildren in Thailand at percentages ranging from 5.6 to 75.1% [8–13]. The predominant STH infection type varies by geographical region. For decades in southern Thailand, the most prevalent parasite infection among all age groups was hookworms [14, 15]. During the 1980s, hookworm infection prevalence in southern Thailand reached 76%. At that time, treatment with a single dose of mebendazole was implemented through a national program [16]. Twenty years later, in 2002, along with better knowledge of personal health and hygiene and more accessible health services, the hookworm infection prevalence among children decreased to 21.1% [17]. Currently, due to rapid urbanization in Thailand, parasitic infections are thought to be further decreasing and have been given relatively little attention by the national government.

Although several studies have been conducted on the distribution and prevalence of intestinal parasitic infections in southern Thailand, epidemiological information on STHs and other intestinal parasitic infections is lacking for several remote areas. Therefore, this study is aimed at assessing the prevalence of STH and other intestinal parasitic infections among schoolchildren in Nopphitam District and identifying potential risk factors for infection or transmission in the study area.

## Methods

### Study design and setting

A cross-sectional study was conducted from January to March 2016 at twelve primary schools in Nopphitam District, Nakhon Si Thammarat Province in southern Thailand. Nopphitam District is located approximately 780 km south of the Thai capital of Bangkok and 80 km from the city of Nakhon Si Thammarat. The average temperature is 27.1 °C, with a low of 25.8 °C in January and a high of 29.3 °C in May. Annual rainfall is 1454.3 mm (Climatological Center, Thai Meteorological Department, Annual Report 2015). The Thailand Department of Provincial Administration estimates that the total population of these subdistricts was 33,183 in 2015. Although Nakhon Si Thammarat Province has been rapidly urbanized in the past 10 years, Nopphitam District has remained rural with only one local public hospital and twelve primary schools. The main access road is non-asphalt, while the other roads are asphalt. Most land is used for farming and rubber plantations.

### Study population and sample size

The study population consisted of children from 7 to 12 years of age attending elementary schools in Nopphitam District. The sample size was determined using the single proportion population formula:

$$ {\mathrm{Z}}^2\ \mathrm{p}\ \left(1-\mathrm{p}\right)/{\mathrm{d}}^2 $$

where p = prevalence of intestinal parasites from a previous study, d = margin of error, and Z = the value from a standard normal distribution corresponding to the desired confidence level, which equals 1.96 for a 95% CI. This formula was calculated based on a prevalence rate of 24.1% from a previous study [17], with a margin of error of 0.05 and a confidence level of 95%. The appropriate sample size was determined to be 286. We assumed that the final sample size would be reduced by 5% due to incomplete data and thus aimed for a sample size of 300. Students who had been treated for intestinal helminthiasis within 1 month of the collection date, declined participation, or could not pass stool on the collection date were excluded from the study. Finally, using class rosters from twelve primary schools as the sample frame, the sample was selected using a systematic random sampling technique.

### Questionnaire survey

A structured questionnaire (Additional file 1) was developed and used to collect demographic data (i.e., age, gender, and education level) and information on possible risk factors (i.e., handwashing before meals, eating raw meat, etc.). The questionnaire was administered by two trained interviewers who directly interviewed the participating schoolchildren.

### Stool sample collection and laboratory procedure

All children were given oral instructions for handling and collecting fecal samples. Each student received a clean plastic container on the day before specimen collection. A single fresh stool sample of approximately 2–10 g was collected from each student. The students returned their stool specimens in labeled containers. Each specimen was checked for correct labeling, quantity, and collection procedure and transported immediately to the parasitology laboratory of the School of Medicine, Walailak University. Part of each stool specimen was processed immediately using a direct microscopic technique with saline and iodine solution to detect protozoan trophozoites, cysts, oocysts, and helminth eggs. The remainder of each stool specimen was analyzed by formalin-ethyl acetate sedimentation concentration, which increases the chances of detecting parasitic organisms in small numbers [18]. Briefly, 1 g of stool sample was suspended in 5 ml of 10% formalin, sieved with cotton gauze, and transferred to a 15-ml conical tube, which was then centrifuged for 10 min at 500×g. Following this, 8 ml of 10% formalin and 4 ml of ethyl acetate were added to the sediment, which was shaken vigorously in an inverted position for 30 s and centrifuged for 10 min at 500×g. Subsequently, the supernatant was discarded, and the remaining sediment was observed under light microscopy at 10× and 40× objectives to detect the eggs and larvae of helminths and cysts and the trophozoites of protozoan parasites. The parasites were morphologically diagnosed under the Centers for Disease Control and Prevention diagnostic reference [18]. Iodine solution was used to detect and identify cysts of protozoan parasites. The modified Kato-Katz technique was also performed to determine the STH infection intensity [19]. Eggs were quantified by species and calculated as eggs per gram (EPG) of stool. According to WHO recommendations, the hookworm infection intensities were classified as light-intensity (1–1999 EPG), moderate-intensity (2000–3999 EPG), and heavy-intensity (≥4000 EPG), respectively. [20]. To eliminate observer bias, each stool sample was examined by two trained senior medical laboratory technologists who were uninformed of the study participants’ identification and behavioral risk factors.

### Data analysis

Data were entered, cleaned, and analyzed using IBM SPSS Statistics for Windows, Version 23.0. Armonk, NY: IBM Corp. Median & interquartile ranges (IQR) for continuous variables and proportions for categorical variables were computed. A mixed effect logistic regression analysis was used to assess the association of independent variables with hookworm infections. The hierarchical structure of the model was the twelve different schools, which were used as random effects in order to account for clustering. Crude odds ratio (COR) of the binary outcome variable (with and without hookworm infection) for each independent variable was assessed by univariable analysis. All variables with *P*-values less than 0.05 in the univariable analysis were analyzed using multivariable analysis to adjust for possible confounders. The final model was interpreted by the adjusted odds ratio (AOR) with a 95% confidence interval (CI). Differences were considered statistically significant when the *P*-value was less than 0.05.

## Results

### Sociodemographic characteristics

A total of 299 participants between 7 and 12 years of age comprised the study sample. The median age was 9 years (IQR: 8–11), with 53.8% of participants (161/299) less than 10 years of age. Males represented 57.5% (172/299) of enrolled participants (Table 1).

**Table 1:** Sociodemographic characteristics of primary schoolchildren in Nopphitam District, Nakhon Si Thammarat Province, Thailand, 2016

Characteristics		Number	Percentage
Gender			
Male		172	57.5
Female		127	42.5
Age group (years)			
7–9		161	53.8
10–12		138	46.2
Level of education (grade)			
1		36	12.0
2		59	19.7
3		66	22.1
4		64	21.4
5		23	7.7
6		51	17.1
Religion	Buddhism	299	100.0

### Prevalence of intestinal parasitic infections

**Fig. 1:**
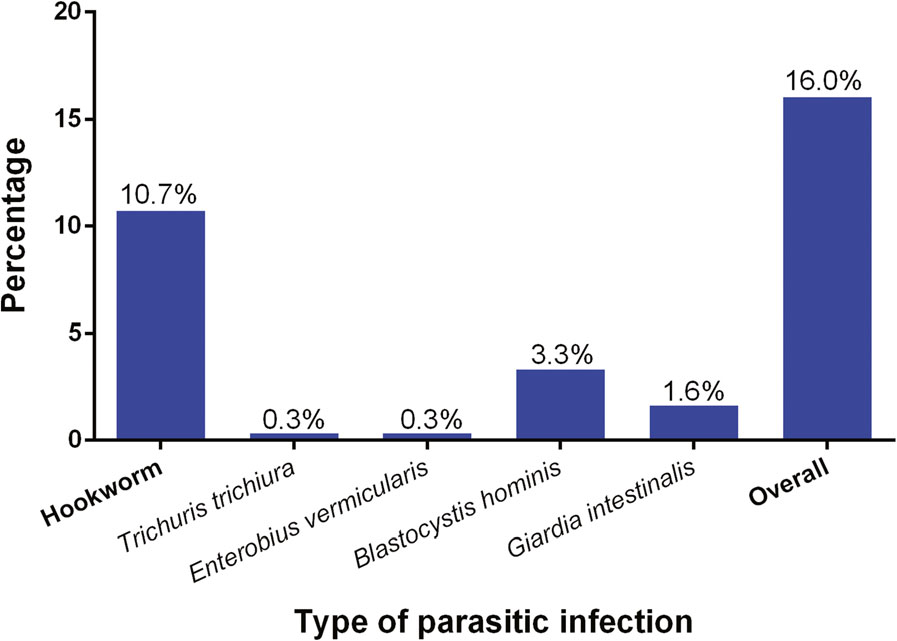
Prevalence of parasitic infections among primary schoolchildren in Nakhon Si Thammarat, southern Thailand, 2016

Of the 299 stool specimens examined, the overall parasitic infection prevalence was 16% (48/299). Hookworms were the predominant parasites, with 32 infection cases (10.7%). The prevalence of intestinal parasitic and protozoal infections is shown in Fig. 1. While most positive cases were single infections, four children (1.3%) had double infections: two had *G. intestinalis* and hookworm infections; one had *B. hominis* and hookworm infections, and one had *T. trichiura* and hookworm infections. The median EPGs of the hookworms and *T. trichiura* were 4550 (IQR: 40–32,360) and 92, respectively. Most hookworm infections (30 of 32) were light-intensity; however, two children had heavy-intensity infections.

### Associations of independent variables with hookworm infections

The study results indicated that hookworm was the most prevalent infection; therefore, we assessed the association between hookworm infections and several potential predictor variables to determine risk factors for the infection. When classified by age, the prevalence of intestinal parasitic infections in children aged 10–12 years was higher (15.2%) than for those aged 7–9 years (6.8%) (COR = 2.2, 95% CI: 1.1–4.7, *P* = 0.037). Children who did not regularly wash their hands before meals were more likely to be infected with hookworms than those who did wash (COR = 2.2; 95% CI: 1.0–4.6, *P* = 0.046). In addition, all 32 children with hookworm infections reported eating fresh vegetables. Furthermore, children who practiced open defecation and played on the ground were 1.6 and 1.8 times more likely, respectively, to have hookworm infections than children without these risk factors. However, these associations were not statistically significant (COR = 1.6; 95% CI: 0.5–5.2, *P* = 0.398 and COR = 1.8; 95% CI: 0.3–12.5, *P* = 0.539, respectively) (Table 2).

**Table 2:** Factors associated with hookworm infection among primary schoolchildren in Nopphitam District, Nakhon Si Thammarat Province, Thailand, 2016

Variables	Number (%)	No. positive (%)	No. negative (%)	COR^a^	95%^c^ CI	*P*-value	AOR^b^	95%^c^ CI	*P*-value
Age (years)									
7–9	161 (53.8)	11 (6.8)	150 (93.2)						
10–12	138 (46.2)	21 (15.2)	117 (84.8)	2.2	1.1–4.7	0.037*	2.3	1.1-4.9	0.030*
Sex									
Male	172 (57.5)	20 (11.6)	152 (88.4)						
Female	127 (42.5)	12 (9.4)	115 (90.6)	1.2	0.6–2.6	0.580			
Handwashing before meals									
Yes	224 (74.9)	19 (8.5)	205 (91.5)						
No	75 (25.1)	13 (17.3)	62 (82.7)	2.2	1.0–4.6	0.046*	2.3	1.1-4.8	0.037*
Drinking water source									
Bottled water	94 (31.4)	8 (8.5)	86 (91.5)						
Tap water	106 (35.5)	19 (17.9)	87 (82.1)	2.2	0.9–5.4	0.069			
Rain	36 (12.0)	1 (2.8)	35 (97.2)	0.5	0.1–2.7	0.408			
Water filter	63 (21.1)	4 (6.3)	59 (93.7)	0.8	0.2–2.6	0.685			
Eating raw meat									
No	110 (36.8)	10 (9.1)	100 (90.9)						
Yes	189 (63.2)	22 (11.6)	167 (88.4)	1.4	0.6–3.1	0.453			
Eating fresh vegetables									
No	17 (5.7)	0 (0)	17 (100.0)						
Yes	282 (94.3)	32 (11.3)	250 (88.7)	4.4	0.2–77.9	0.314			
Open defecation									
No	272 (91.0)	28 (10.3)	244 (89.7)						
Yes	27 (9.0)	4 (14.8)	23 (85.2)	1.6	0.5–5.2	0.398			
Contact with domestic animals									
No	24 (8.0)	4 (16.7)	20 (83.3)						
Yes	275 (92.0)	28 (10.2)	247 (89.8)	0.5	0.2–1.7	0.297			
Wearing shoes outside the house									
Yes	137 (45.8)	17 (12.4)	120 (87.6)						
No	162 (54.2)	15 (9.3)	147 (90.7)	0.7	0.4–1.5	0.419			
Playing on ground									
No	18 (6.0)	1 (5.6)	17 (94.4)						
Yes	281 (94.0)	31 (11.0)	250 (89.0)	1.8	0.3–12.5	0.539			

*Significant association

^a^ COR: crude odds ratio by univariable analysis

^b^ AOR: adjusted odds ratio by multivariable analysis

^c^ CI: 95% confidence interval

Multivariable analysis also indicated that age and handwashing before meals were significantly associated with hookworm infections. Children aged 10–12 years were more likely to have hookworm infections than those aged 7–9 years (AOR = 2.3, 95% CI: 1.1–4.9, *P* = 0.030). Children who did not regularly wash their hands before meals were more likely to be infected with STH than children who did practice good hand washing (AOR = 2.3; 95% CI: 1.1–4.8, *P* = 0.037) (Table 2).

## Discussion

We determined the overall prevalence of intestinal parasitic infections among primary school children 7–12 years of age from Nopphitam District, Nakhon Si Thammarat Province in southern Thailand. Of 299 schoolchildren, 48 (16%) were positive for at least one intestinal parasite, including both helminths and protozoa. The observed prevalence of intestinal parasites was higher than the prevalences found in similar reports conducted in other parts of Thailand over the past two decades. Those prevalence rates included 4.24% in central area of Thailand, including Ang Thong, Ayutthaya, and Suphanburi Provinces in 2004 [21]; 13.9% in Pathum Thani Province in 2010 [10] and 5.4% in Phitsanulok Province, in lower northern area of Thailand during 2009 and 2010 [22]. The present study found that intestinal parasitic infection remains a public health problem in southern Thailand compared with other parts of the country. Furthermore, it also indicates that helminths and protozoa are prevalent in these areas. In contrast, the prevalence of overall intestinal parasitic infections observed in this study was lower than in previous studies conducted in southern Thailand using the same diagnostic method (formalin-ethyl acetate sedimentation concentration) used in this study, specifically 75.1% in Narathiwat Province in 2003 [9] and 19.8% in a national survey conducted in southern Thailand in 2009 [15]. Improved environmental and socioeconomic factors may account for the decreased prevalence of intestinal parasites in these areas. These data show that over two decades, the prevalence of intestinal parasitic infections in southern Thailand has decreased but remains a problem.

Hookworms were the most common helminths found in the schoolchildren, which is consistent with previous reports conducted in southern Thailand [15–17]. However, the observed hookworm infection prevalence of 10.7% was lower than reports from a similar study in southern Thailand, which reported a prevalence of 21.1% in 2002 [17], and a national survey, which reported a prevalence of 15.8% in 2009 [15]. The lower hookworm infection prevalence in the present study may be due to differences in urbanization, improved knowledge of personal hygiene, and improved environmental sanitation, for example, clean and safe water supply, and effective human and animal waste disposal. However, further work is needed to detect the health impacts of hookworm infections on children in the studied community.

From the 32 schoolchildren with STH infections, one was coinfected with hookworms and *T. trichiura. Ascaris lumbricoides,* another STH, was not found among these schoolchildren. This low *T. trichiura* infection prevalence and the absence of *A. lumbricoides* are consistent with a previous report by Anantaphruti et al., 2002, which reported a hookworm prevalence of 21.1% and a *T. trichiura* prevalence of 5.4%, with no *A. lumbricoides* [17]. The important factors affecting the prevalence of STH in southern Thailand are tropical climatic conditions and moist soil that is suitable for the survival of parasitic eggs. In this study, more than half of study subjects reported risk behaviors related to STH infections, including walking barefoot (54.2%) and playing on the ground (94%). These data suggest that health education on personal hygiene should be part of the educational curriculum in primary schools in the study area.

The previous control measures for STH infections in the study area were limited to one school, which is a representative of Nopphitam District where the Ministry of Public Health administers regular fecal examinations every 3 years. Testing results of this program revealed less than a 5% prevalence of parasitic infections. Accordingly, Nopphitam District is considered a low-prevalence area by the Ministry of Public Health, and drug treatment is administered on a three-year basis for students in the school. The results of this study reveal that control measures are not universally effective in the study area, as the prevalence of hookworm infections remained as high as 10.7%. Although WHO recommends that mass drug administration (MDA) should be performed once annually when the baseline STH infection prevalence in a community is over 20% [5], we suggest that MDA programs should be considered in the study area even though prevalence did not reach 20%, as this practice is a mainstay of controlling STH infections.

As this study revealed that hookworm was the most common parasitic infection among schoolchildren in the studied community, multivariable analysis identified age and handwashing before meals as statistically significant associated risk factors for hookworm infection. Schoolchildren in the 10- to 12-year-old group had a significantly higher percentage of hookworm infections than those in the 7- to 9-year-old group. This trend was also observed in studies conducted in other countries [21–26]. This may be explained because as children grow older, their outdoor activities increase, thus increasing their STH exposure.

Moreover, schoolchildren who did not regularly practice handwashing before meals were significantly infected with hookworms at a rate 2.3 times higher than those who did wash. Although these findings were not statistically significant, children who practiced open defecation and children who played on the ground had 1.6 and 1.8 times higher risks, respectively, of having hookworm infections than their counterparts. This may be explained because the hookworm’s mode of transmission is not limited to skin penetration but may also occur by the fecal-oral route; thus, handwashing before meals played an important role in acquiring hookworms in our study.

For protozoal infections, the most predominant protozoa were *B. hominis*, with 10 infections (3.3%), and *G. intestinalis*, with 7 infections (2.3%). *B. hominis* has been traditionally regarded as a harmless parasite of humans. However, it can cause symptoms such as abdominal pain, constipation, diarrhea, and irritable bowel syndrome [27, 28]. Five or more *B. hominis* cells per 40× magnification field seen during direct microscopic examination of a stool smear wet mount is considered to cause clinical illness [29]. In this study, all fecal samples with *B. hominis* revealed more than five cells per 40× magnification, hence, this protozoa might cause gastrointestinal symptoms among these children, however, we obtained no information on clinical details. These *B. hominis* infections could result from ingestion of contaminated water or food. Additional study on this protozoal burden is needed. Further, more sensitive tests, such as molecular techniques, should be considered for identifying these protozoa [18]; however, such tests are generally expensive and require skilled technicians.

This study had the following limitations. Each method used in our study has varying sensitivity among STHs. The sensitivities are highest for modified Kato-Katz technique with 63.8%, 82.2% and 59.5% for *A. lumbricoides*, *T. trichiura*, and hookworm, respectively [30]. Secondly, formalin-ethyl acetate sedimentation concentration technique has sensitivities of 56.9%, 81.2% and 53.0% for *A. lumbricoides*, *T. trichiura*, and hookworm, respectively [30]. The lowest sensitivity was noted for the direct microscopy method with 52.1%, 62.8% and 42.8% for *A. lumbricoides*, *T. trichiura*, and hookworm, respectively [30]. Obtaining multiple stool samples is recommended to improve diagnostic sensitivity, especially for hookworm infection [31], hence, using a single-day fecal examination to determine helminth presence was the major limitation of our study. Furthermore, associated risk factor items were restricted to questionnaire data and might not entirely represent all associated factors. Finally, this study was conducted in Nopphitam District located in southern Thailand, which has distinct tropical hot and humid climate compared with other parts of the country, so the results might not be applied to other geographical settings, especially those outside southern Thailand. The strengths of this study include that the parasite detection yield was increased by using combination of three methods, namely, direct wet smears, the formalin-ethyl acetate concentration technique and a modified Kato-Katz technique [32, 33]. Furthermore, these tests yield high specificity ranged from 97.5 to 99.6% [30]. In addition, this study is the first to report parasitic infection prevalence among children in Thailand in the past 7 years.

## Conclusions

Hookworm remains the most predominant STH infection among schoolchildren in rural areas of Nakhon Si Thammarat. Older age group (10–12 years) and inadequate handwashing before meals were statistically associated with hookworm infections. Due to the substantial intestinal parasitic infection prevalence (especially of hookworms), risk factor-related behaviors among schoolchildren, and suitable humidity for STHs in southern Thailand, appropriate control measures should be taken to reduce the prevalence of STH infections in this community, including education on personal hygiene and environmental sanitation and the development of awareness strategies and MDA programs.

## Additional file

Additional files 1: Questionnaire on demographic data and possible risk factors. (PDF 481 kb)

## Abbreviations

STH: Soil-transmitted helminth
EPG: eggs per gram
AOR: adjusted odds ratio
CI: confidence interval
IQR: interquartile range
COR: crude odds ratio
MDA: mass drug administration
WHO: World Health Organization
