# Supplementary material for: Prevalence of intestinal parasitic infections and associated risk factors for hookworm infections among primary schoolchildren in rural areas of Nakhon Si Thammarat, southern Thailand
Source: BMC Public Health. 2018 Sep 14;18:1118. doi: 10.1186/s12889-018-6023-3 (PMC6137929; doi:10.1186/s12889-018-6023-3)
Supplement: Supplementary file 1 — Questionnaire on demographic data and possible risk factors. (PDF 481 kb) [file 12889_2018_6023_MOESM1_ESM.pdf]

Questionnaire

Part 1: General information

1. Code no. ....
2. Gender ❍ 1. Male ❍ 2. Female
3. Age .... years
4. Current grade............... Name of School……………………………………
5. Religion ❍ 1. Buddhism ❍ 2. Christian
   ❍ 3. Islam ❍ 4. Other…………………
6. Pet(s) at home ❍ 1. Dog(s)
   ❍ 2. Cat(s)
   ❍ 3. Other………………….
   ❍ 4. None

Part 2: Personal hygiene and possible risk factors

1) Do you wash hands before meal?

❍ Never/seldom ❍ Regularly

2) Which is your drinking water source?

❍ Tap water ❍ Rain water

❍ Filtered water ❍ Bottled water

3) Do you eat raw /undercooked /uncooked meat?

❍ Never/seldom ❍ Regularly

4) Do you eat fresh vegetables?

❍ Never/seldom ❍ Regularly

5) How do you defecate at home?

❍ Using latrine

❍ Open defecation for example: defecation in the woods/on soil-ground

6) In a week, do you play or contact with pets?

❍ No ❍ Yes

7) Do you wear shoes when going outside the house?

❍ Never/seldom ❍ Regularly

8) Do you play on soil-ground or lawn?

❍ Never/seldom ❍ Regularly

☺Thank you very much☺
